# 

**Published:** 1895-07

**Authors:** 


### CONTENTS—VOL. II., 1895-6.

Artificial Anus.......... 1
American Medical Association.......... 565
Anomalies of Typhoid Fever.......... 163
Acute Rheumatism—Salicylates in.......... 177
Abortion of Typhoid Fever.......... 171
Analgesia and Sedation.......... 322
Acute Bronchial and Pneumonic Diseases of Children—Treatment of.......... 350
Approching Insanity—Signs, etc., of.......... 409
Antisepses and Antiseptics.......... 433
Alcohol as a Factor in Crime.......... 449
Alcohol, the Demon.......... 453
A Plea for More Efficient Medical Laws.......... 490
A Plea for Reform in Criminal Jurisprudence.......... 597
Address—President's T. S. M. A.......... 622
"A Convenient Memory" Indeed.......... 702
A Fishy Novel, by a Texas Doctor.......... 247
A Flop Extraordinary.......... 695
A State Board of Health for Texas.......... 691
A Texas Home for Epileptics.......... 251

Bennett, Dr. T. J.......... 64
Bite of Spider, or Spider Bite.......... 111
Bicycle—The, from Surgical Standpoint.......... 140
Bicycling for Women.......... 142
Bladder—Retroversion of.......... 540
Bloodletting in Pneumonia.......... 437
Brittain, Dr. F. B.......... 619
Book Notices.........42, 95, 154, 212, 336, 400, 465, 526, 279, 650
Brazos Valley Medical Association.......... 689
British Medical Association—Transactions of.......... 239

Clinical Investigation of Tannigen.......... 17
Clinical Microscopy.......... 53
Colorado State Medical Association.......... 79
Cummings, Dr. Jos.—Death of.......... 86
Castration the Remedy for Crime.......... 133
Castration for Crime Not Justifiable.......... 547
Castration for Crime—as Preventive and Curative Treatment.......... 498
Cerna, Dr. David.......... 549
Circular—The Committee's to Physicians.......... 590
Cycling and Heart Disease.......... 193
Cataract Operation—Tyner's.......... 200
Cook, Dr. E. T.—Letter from.......... 359
Congestion..........362, 375
Calhoun, Dr. P. F.......... 433
Criminal Jurisprudence—Reform in.......... 597

Coleman, Dr. B. C.—Address by as President.................. 622
Circular—A to Physicians.......................................... 652
Correspondence........................75, 76, 113, 547, 359, 442, 503
Cronin, Dr. P. H.................................................. 225
Clinical Cases.................................................... 225
Cholelithiasis—Surgical Treatment of.............................. 228

DuPre, Dr. D...................................................... 60
Decline and Falling Off—The....................................... 84
Death of Dr. Cummings............................................. 86
Denman, Dr. A. N.................................................. 113
Discussion on Erysipelas.......................................... 124
DeCausey, Dr. M................................................... 547
Doctors—A Warning to.............................................. 581
Dr. Sam........................................................... 192
Diphtheria and Sewer Gas.......................................... 356
Duncan, Dr. J. F.................................................. 356
Daniel, Dr.—One on................................................ 458
Discussion on Dr. Olive's Paper................................... 480
Dermatology—Section on............................................ 510
Daniel, Dr. F. E.................................................. 597
Department of Practice of Medicine.............73, 167, 195, 369

Evans, Dr. J. H................................................... 32
Eclampsia Puerperal............................................... 70
Erysipelas—Discussion on.......................................... 124
Evolution and Heredity............................................ 168
Extra Uterine Pregnancy—Report on two cases of.................... 283
Ellis, Dr. G. Manning............................................. 288
Epileptics—State Care of..........................325,363, 506, 511
Ectopic Gestation—Report of Cases................................. 421
Epileptics—A Texas Home for....................................... 251

Fecal Fistulæ, and Artificial Anus................................ 1
Fractures Ununited................................................ 27
Fibroid Uterus, Hysterectomy for.................................. 32
Forbes, Dr. C. J.................................................. 163
Frazier, Dr. J. W................................................. 498
Fort Worth Meeting, The........................................... 516
Forceps, a New Uterine............................................ 235
For "Durin' the War".............................................. 693

Goitre, Operations for, 13 Cases.................................. 33
Gangrene.......................................................... 60
Gonorrhœa, Treatment of, etc...................................... 179
" in Women, Treatment of, etc..................................... 211

Hysterectomy for Fibroid Uterus................................... 32
Hadra, Dr. B. E................................................... 33
Hold Out Your Tongue.............................................. 36
Hæmorrhoids, Modern Treatment of.................................. 64
Hemorrhagic Malarial Fever........................................ 105
Humerus, Resection of............................................. 545

Humerus, Dislocation of, at Shoulder........................... 159
Hill, Dr. H. B........................................................ 159
Hot Springs, Ark.................................................... 359
Hargis, Dr. J. W..................................................... 504

Intermittent Fever, Treatment of............................. 257
Importation of Cattle, Order Concerning..................... 245
Iris, Melanoma of the............................................. 221
Inflammation of Uterine Appendages.......................... 35
Indian Hemp Drug Commission.................................. 138
Intestinal Obstruction, Maydl's Operation in................ 347
Intervention in Pregnancy, Complicated, etc................ 354
Insanity, Approaching, Signs and Symptoms of............. 409
Insane, State Care of the, etc.................................. 613

Jones, Dr. I. J....................................................... 105
Johnson County Medical Society............................... 566

Kunkler, Karl, Dr.................................................. 17

Karnes' (Dr.) Case................................................ 76

Lamphear, Dr. Emory.............................................. 70
Love, Dr. F. S....................................................... 421
Learned Ass, The................................................... 456

Menopause, The Complications, etc............................ 9
Miasmatic Paralytic Fever........................................ 38
Microscopy, Clinical............................................... 53
Medico-Legal Congress............................................ 77
Medical Liberalism................................................. 79
Mandrakes, Dr. Merriman on..................................... 81
Malarial (hemorrhagic) Fever.................................... 105
Mental Therapy..................................................... 533
Medical Profession of Texas, To the........................... 562
Medico-Legal Society, Chicago.................................. 564
Medical Journal (the) the Post-Graduate Instructor......... 572
Medical Society, Tri-State........................................ 302
Medical Association, West Texas................................ 319
Milliken, Dr. Sam.................................................. 323
Maydl's Operation, Remarks on.................................. 347
Medical Men in Politics........................................... 373
Medical News and Miscellany.....................................
...............41, 87, 150, 203, 329, 380, 460, 518, 582, 648, 260
Melanoma of the Iris.............................................. 221
Mullins, Dr. Jos. A................................................ 221
Morris, Dr. Robt. T................................................ 225

Narrow escape, a................................................... 41
New Woman, the, and Bicycle.................................... 135
Norsworthy, Dr. C. S............................................... 392
National Conference State Boards of Health.................. 246
Notes on Some of the Newer Methods of Treatment of
Nervous and Mental Disease.................................... 659

Operations for goitre, 13 cases........ 33
Ophthalmology, progress in........ 121, 297
Orr, Dr. James........ 350
One on Dr. Daniel (and Thompson)........ 458
Olive, Dr. N. A........ 9, 473
Our Birth-Day........ 702

Peeples, Dr., miasmatic paralytic fever........ 75
Peeples, Dr. D. L........ 40
Puerperal eclampsia........ 76
Pope, Dr. T. A........ 95
Progress in ophthalmology........ 121
Pugh, Dr. W. W........ 545
Paulus, Dr. A. D., death of........ 197
Prescriptions, a few selected........ 209
Paralysis, pseudo-muscular hyperthrophic........ 288
Pericarditis, suppurating........ 292
Paschall, Dr. Frank........ 354
Pregnancy, intervention in........ 354
Pasteur and his discoveries........ 371
Practice acts, the, in Texas........ 382
Patella, fracture of........ 428
Perkins, Dr. A. N........ 437
President (T. S. M. A.) address........ 622
Pseudo chancre........ 244

Regulating the practice........ 37
Richmond, Dr. W. T........ 111
Resection, that........ 113
Richmond Academy, Med. and Surgery........ 118, 237, 559
Rational Remedy, the only........ 158
Retroversion of Bladder........ 540
Resection of Humerous........ 545
Retrodeviations of Uterus........ 566
Rules for the Surgeon........ 573
Rheumatism, Successful treatment of........ 320
Removal of Spleen........ 358
Regulate the Practice, To........ 455

State Boards of Health, National Conference of........ 246
Stuart, Dr. J. R........ 27
"Sossige," The........ 36
Smith, Prof. A. J........ 73, 167, 295, 369
Spider bite or bite of spider........ 111
Sufferers who long to die........ 139
Smith, Dr. M. M........ 533
Spohn, Dr. A. E........ 540
Small publishers, death to........ 570
Salicylates in acute rheumatism........ 177
Sullivan, Dr. Jno. J........ 323
Spleen Removal........ 358
Sociology and Criminology........ 365

Southwestern Medical Record, The.................................. 460
Spohn, Dr. E. A., Letter from........................................ 503
State Care of Epileptics.......................325, 363, 506, 511, 613
Society Notes.......................118, 237, 302, 365, 446, 507, 552
Some Cases of Osteomyelitis........................................... 675
Some of the Mistakes of Surgical Gynecology................. 680

Thompson, Prof. J. E.................................................... 283
Tannegin, Clinical Investigation of, Properties of............ 17
Todd, Dr. F. C...................................................121, 297
Therapeutics, Department of........................................ 549
Texas State Medical Association .......................378, 447, 552
Texas State Medical Association, Fort Worth meeting of... 516
Tuberculosis, Alleged New Treatment for........................ 575
Typhoid Fever, an Analysis of...................................... 163
Typhoid Fever, Abortion of........................................... 181
Typhoid Fever, Is It Contagious?.................................. 504
Taka Diastase............................................................. 188
Tyner's Cataract Operation........................................... 200
Tendon Grafting........................................................... 323
Texas Medical College; The.......................................... 324
Terrell Med. Society, Proceedings of..................368, 446, 507
Therapeutics, Department of........................................ 439
Things Observed in New York (letter)........................... 442
Texas S. M. A., 28th Annual Meeting of......................... 631
Typhoid Fever, Diagnosis and Treatment of.................... 619
Taylor, Dr. Hugh M....................................................... 228
Turpin, Dr. T. J............................................................ 235
The Doctor and Politics................................................ 255
The Keynote to Success................................................ 256
Treatment of Intermittent Fever.................................... 257
The North Texas Medical Association............................ 686
The East Texas Medical Association............................. 690
The Texas Medical News.............................................. 699

Ununited Fractures....................................................... 27
Uric Acid Diathesis; Cause and Treatment of.................. 473
Uterine Appendages; Inflammation of............................ 35

Vanity of Vanities........................................................ 254

Walker, Dr. W. W.......................................................... 76
Water Question; The..................................................... 143
Water, Purification by Filtration .................................. 568
Water Purification........................................................ 201
Wile, Dr. W. C............................................................. 320
Wooten, Dr. T. D.......................................................... 347
Wallace, Dr. D. R.......................................................... 409
Weller, Dr. C. O........................................................... 428
Woldert, Dr. E. A.......................................................... 490
White, Dr. F. S............................................................. 613

Young, Dr. H. H............................................................ 53
Yandell, Dr. W. M......................................................... 356

